# The Potential Possibility of Symptom Checker

**DOI:** 10.15171/ijhpm.2017.41

**Published:** 2017-04-05

**Authors:** Tomohiro Morita, Abidur Rahman, Takanori Hasegawa, Akihiko Ozaki, Tetsuya Tanimoto

**Affiliations:** ^1^ Department of Internal Medicine, Soma Central Hospital, Soma, Japan.; ^2^ Department of Medicine, Shaheed Suhrawardy Medical College & Hospital, Dhaka, Bangladesh.; ^3^ Health Intelligence Center, The Institute of Medical Science, The University of Tokyo, Minato-ku, Japan.; ^4^ Department of Surgery, Minamisoma Municipal General Hospital, Fukushima, Japan.; ^5^ Department of Internal Medicine, Jyoban Hospital of Tokiwa Foundation, Fukushima, Japan.

## Dear Editor,

The access to medical care is unacceptably low worldwide in spite of the increasing demand for medical care due to population aging and increasing burden of non-infectious diseases. The probability of patients receiving at least one medicine for secondary prevention of cardiovascular disease was 19.8% in low-income countries, 30.7% in low- and middle-income countries, and 54.9% for upper-middle-income countries.^1^ Especially, low- to middle-income countries are seriously in short of professional medical staff. For example, in Bangladesh, the doctor-population and the doctors-nurse ratio is 1:12 690 and 2.5:1, respectively, which is among the lowest group in the world.^2^ In such resource-limited countries, patients cannot access to medical care in a timely manner. Thus, the tool to help patients with self-diagnosis and self-triage is urgently needed to mitigate the shortage of medical care resources.

Symptom checkers are algorithm-based tools for self-diagnosis and self-triage. The increasing access to the Internet enabled these kinds of web-based healthcare service including symptom checkers. Generally, symptom checkers make a diagnosis of a disease based on the data on the prevalence of disease and its sensitivity and specificity of symptoms. We would like to introduce the possible benefit of symptom checkers on public health.

One of the main functions of symptom checkers is to assist with triage, which is the same function of community health workers (CHWs) in resource-limited countries. In Bangladesh, community health workers trained by NGOs contribute to promote public health, which has been proved effective such as in reducing mortality among infants in rural areas.^3^ However, quality control or education cost for health workers are now becoming social issues.^4^ It is sometimes difficult or impossible for the doctors or health workers to maintain the quality of the treatment since they do not analyze the patient’s symptoms with the medical database or due to lack of their up to date knowledge. They just do some specific tests to assess the health condition of the patients and using their sport observation they draw the conclusion and suggests medicine for the patient’s remedy from diseases or illness. Symptom checkers could help CHWs to triage patients. In the future, symptom checkers would help keeping up the quality of primary care delivered by CHWs in resource-limited countries.

Though, prior researches suggest that symptom checkers may be less effective than physicians in diagnostic accuracy for now,^5^ to conclude the superiority of doctors to symptom checkers might be overhasty. This is because diagnostic accuracy of symptom checkers can be improved after appropriate feedback. One reason of low diagnostic accuracy is because there is no accurate database of these information for all diseases. However, with the feedback of the data of diagnoses along with patients’ symptoms reported by doctors, the symptom checkers can update their incorporated database, making the diagnosis closer to doctors’ diagnosis than before. After doctors’ feedback, the updated database of symptom checkers can provide useful information for clinical education (eg, sensitivity and specificity of symptoms for diseases, prevalence of diseases). Therefore, symptom checkers could be more useful and helpful tools for medical staff.

Symptom checkers can not only be the beneficial tools for doctors but also provide the access to health care in low resource settings such as in rural areas or developing countries. We doctors should concern that our active cooperation with symptom checkers can contribute to improve public health.

## Ethical issues

Not applicable.

## Competing interests

Authors declare that they have no competing interests.

## Authors’ contributions

TM and TH developed the concept of the letter. TM and AR collected data. TM drafted the manuscript and all authors contributed substantially to its revision.

## Authors’ affiliations

^1^Department of Internal Medicine, Soma Central Hospital, Soma, Japan. ^2^Department of Medicine, Shaheed Suhrawardy Medical College & Hospital, Dhaka, Bangladesh. ^3^Health Intelligence Center, The Institute of Medical Science, The University of Tokyo, Minato-ku, Japan. ^4^Department of Surgery, Minamisoma Municipal General Hospital, Fukushima, Japan. ^5^Department of Internal Medicine, Jyoban Hospital of Tokiwa Foundation, Fukushima, Japan.

## References

[1] Semigran HL, Levine DM, Nundy S, Mehrotra A (2016). Comparison of Physician and Computer Diagnostic Accuracy. JAMA Intern Med.

[2] Semigran HL, Linder JA, Gidengil C, Mehrotra A (2015). Evaluation of symptom checkers for self diagnosis and triage: audit study. BMJ.

[3] Prince MR. Health Status of Bangladesh 2015. http://www.theindependentbd.com/printversion/details/24301. Accessed December 19, 2016. Published 2015.

[4] Mercer A, Khan MH, Daulatuzzaman M, Reid J (2004). Effectiveness of an NGO primary health care programme in rural Bangladesh: evidence from the management information system. Health Policy Plan.

[5] Rowe AK, de Savigny D, Lanata CF, Victora CG (2005). How can we achieve and maintain high-quality performance of health workers in low-resource settings?. Lancet.

